# Saponin Stabilization via Progressive Freeze Concentration and Sterilization Treatment

**DOI:** 10.3390/molecules26164856

**Published:** 2021-08-11

**Authors:** Wan Nur Aisyah Wan Osman, Dineshraj Selvarajah, Shafirah Samsuri

**Affiliations:** 1 Chemical Engineering Department, Universiti Teknologi PETRONAS, Seri Iskandar 32610, Perak, Malaysia; wan_19001650@utp.edu.my (W.N.A.W.O.); dineshraj_23725@utp.edu.my (D.S.); 2 HICoE-Centre for Biofuel and Biochemical Research, Institute of Self-Sustainable Building, Universiti Teknologi PETRONAS, Seri Iskandar 32610, Perak, Malaysia

**Keywords:** biopesticides, progressive freeze concentration, saponin, stability analysis

## Abstract

Saponin is a biopesticide used to suppress the growth of the golden apple snail population. This study aims to determine the stabilized conditions for saponin storage. The maceration process was used for saponin extraction, and for saponin concentration, progressive freeze concentration (PFC) was used. Afterwards, stability analysis was performed by storing the sample for 21 days in two conditions: Room temperature (26 °C) and cold room (10 °C). The samples kept in a cold room were sterilized samples that undergo thermal treatment by placing the sample in the water bath. The non-sterilized samples were kept in room temperature condition for 21 days. The results showed that saponin stored in the cold room (sterilized sample) has low degradation with higher concentration than those stored at room temperature in stability analysis with the highest saponin concentration (0.730 mg/mL) at a concentration temperature of −6 °C and concentration time of 15 min. The lowest saponin concentration obtained by saponin stored at room temperature (non-sterilized sample) is 0.025 mg/mL at a concentration temperature of −6 °C and concentration time of 10 min. Thus, the finding concluded that saponin is sensitive to temperature. Hence, the best storage condition to store saponin after thermal treatment is to keep it in a cold room at 10 °C.

## 1. Introduction

*Oryza sativa* is the scientific name for rice. It is widely consumed as a staple food by over 2 billion people in Asia, which shows its crucial role in providing food security [1]. Therefore, there is a pressing need to boost the output of irrigated paddy fields to increase rice production with a rising population. However, Malaysia’s warm and humid weather attracts several pests that severely affect the harvest of paddy fields, resulting in a drop in overall rice yields. About 800 species of herbivorous insects inhabit the ecosystem [2]. According to Matteson [3], insect and pest vitiation is crucial for minimal rice harvesting in the tropical Asian region. Additionally, various types of infections caused by fungi, bacteria, and viruses affect rice’s growth and yield. 

For instance, one of the most famous virus outbreaks that have affected rice production since 2001 is the southern rice black-streaked dwarf virus (SRBSDV), first discovered in Yangxi County, Guangdong Province, China [4,5]. Nevertheless, the disease had spread to northern Vietnam in 2009 in which about 103,784 acres of rice were destroyed, and the next following year, more than 148,263 acres of rice were infected. Meanwhile, southern China reported about 741,316 and 3,212,369 acres of rice were infected in 2009 and 2010, respectively [6,7]. Since then, various methods have been used to control SRBSDV, but these two countries reported about 1,729,737 and 1,235,526 acres of rice infected in 2011 and 2012, respectively [8]. In a recent study, D. Wang et al. [9] proposed an antiviral agent known as dufulin, proven effective against plant viruses. It would activate the systemic acquired resistance (SAR) in a plant, reducing the tendency to be infected by any virus. Authors claimed that dufulin only targeted the virus and protected the plant from being infected by the SRBSDV virus, and no adverse effects of dufulin were found on the plant [9].

In addition to virus’ infection, insect and pest vitiation is also a limitation that needs to be overcome for agriculture purposes. Golden apple snail (*Pomacea canaliculata*) has unexpectedly developed into a pest of rice. The snail was introduced intentionally into Asia in 1980 without prior studies on economic benefits or its effect on the ecosystem [10]. It is expected to be cultivated as a high-protein food source for local consumption and export commodity for high-income countries. Still, many snail farming projects were abandoned when the market response was inadequate. In many instances, the snails invaded Asian rice systems with losses of millions of dollars [11]. Hand-picking, use of ducks [12] and fish [13] as biological control agents, and appropriate water control, including occasional field drainage and maintenance of low water levels, are effective in the management of snail population [11]. However, these methods require many workforces and are helpful only in countries where the labour costs are still low. Meanwhile, another approach commonly practised is chemical spray due to simple handling [14].

Consumption of paddy that has been sprayed with chemical insecticides could end up in health-related problems to humans. The continued use of the chemical causes environmental pollution, public health effects, domestic animal poisonings, contaminated products, destruction of beneficial natural predators and parasites, and pesticide resistance in pests [15]. In a recent study, Chen et al. [16] introduced herbicides in microcapsules to replace chemical insecticides. They designed and synthesized herbicide microcapsule systems with high retention rates with the excellent sustained-release ability for the use in paddy field. Many studies agreed that herbicides microcapsules have a controlled release ability that could prevent herbicides from leaking (either via evaporation or degradation), hence, could increase the efficiency of herbicidal and, at the same time, minimize the toxicity [17,18,19,20].

On the other hand, another strategy was also introduced, which is the use of biopesticides. Biopesticides offer a technically feasible and environmentally acceptable approach for controlling agronomically essential insects, including snails. They also can enhance plant productivity and improve plant physiology [21]. The most common benefits of biopesticides are biodegradable, less toxicity, produce lower pesticide residue, and avoid pollution problems associated with chemical pesticides [22].

Moreover, it was also reported that plant metabolites were acknowledged as protection against snails [23,24]. More than 1500 species of plants had been evaluated for potential molluscicidal activity, and most of them are the family of *Theaceae*, *Meliaceae*, *Apocynaceae*, *Euphorbiaceae*, *Leguminosae*, *Phytolaccaceae*, *Solanaceae,* and *Rutaceae* [25]. Molluscicidal activity is a poison and toxic effect that causes the slugs (snails) to secrete vast amounts of mucus, resulting in death. The plant species that have the highest molluscicidal activity is known as *Theaceae* family. One related study had developed the extracts from camellia (one of the species from *Theaceae* family) as molluscicides. The main active compounds found in camellia are saponins [26]. 

Saponins are natural tensoactive found in plants, in which two significant types are triterpenoid and steroidal saponins [27,28]. Both types of saponin have the most similarities in terms of their properties. Only that, they are different in terms of their structure where triterpenoid saponins have pentacyclic molecules (synthesized from isoprene) and tend to be acidic in pH (pH < 7), while steroidal sapnonin have tetracyclic molecules (synthesized from acetyl coenzyme A) and tend to be neutral in pH (pH 7) [29].

During an earlier research on saponin, it was mainly used in the food industry as it had been acknowledged as the antinutritional factor [30]. It was proven that saponin’s food sources could provide health benefits upon consumption, particularly reducing cholesterol [31] and anti-cancer properties [32]. Examples of saponin sources of food contributing to health benefits are garlic [33] and soybeans [34]. Matsuura [33] claimed that the saponin from garlic reduced cholesterol concentrations and levels. This finding was aligned with Kim et al. [31], where saponin contents in food act as cholesterol reduction properties.

Meanwhile, Kerwin [34] found that saponin from soybean or soy-based products has high potential as a cancer prevention alternative. A similar result was found by Gurfinkel and Rao [32], where they mentioned that saponin in food acts as an anti-cancer property. However, saponin’s uses in the food industry were limited due to its bitter taste [35]. J. Liu and Henkel [36] investigated the feasibility of saponin for the application of herbal medicines. Their study mentioned that saponin was the key ingredient in traditional Chinese medicine (TCM) remedies due to its chemical composition and treatment philosophy of TCM. On the other hand, saponin was also valuable for other industrial purposes. Martin and Briones [37] have proven that saponin was helpful for different applications. Their study explained that the bark of *Quillaja Saponaria* tree (significant sources of triterpenoid saponins) had been used as an emulsifier in the food industry and foaming agent, and in the beverages industry as a wetting agent in photography application for about decades.

Since saponin has a high potential to be commercialized in many industrial applications, the saponin extraction process either develops new process strategies or evaluates the existing technologies to increase the saponin extraction process’s performance. In this study, the main focus is saponin extraction for biopesticides application. The use of saponin as biopesticide causes the mortality of golden apple snails (*Pomacea canaliculate*) at paddy fields [27]. Moreover, plants produce saponin as pathogenic agents since it possesses immune-stimulating activities, antimicrobial, anti-cancer, anti-fungal, antiviral, and anti-inflammatory properties [38]. Examples of leaves that have saponin are *monochorea vaginalis*, *furcraea selloa var. marginata*, *furcraea gigantea*, spent tea leaves, and tea factory waste [38,39,40].

Although companies of crops and farmers are successfully adopting biopesticides, there is still an issue that needs to be improved: Biopesticides shelf life [41]. Biopesticides can improve plant physiology by destroying golden apple snails but cannot make them stay long during storage. It is necessary to stabilize and store them with only minimal alteration of their biological activity. To maintain ingredients of biopesticides to be active and stable, methods to destroy unwanted bacteria and remove water from biopesticides should be applied.

To do this, PFC is proposed to concentrate and preserve the saponin. Miyawaki and Inakuma [42] recently reviewed the performance of PFC for various applications, such as for the concentration of natural flavours, fruit juices, fermented alcoholic drinks, coffee and tea extract. They claimed that the concentrate via PFC retains the originality of compounds even after the concentration process. Hence, the interest in using PFC for many concentration processes has been growing rapidly these few years. The sterilization to kill the unwanted bacteria that accumulate on the biopesticides was done. The removal of unwanted bacteria will enhance the shelf life of biopesticides. Therefore, this study aims to determine the stabilized condition for saponin via PFC and sterilization. Afterwards, the stability analysis was performed by storing the sample in two conditions: Room temperature (26 °C) and cold room (10 °C). Lastly, a comparison between the stability of sterilized and non-sterilized concentrated saponin was made to determine the best storage condition to store saponin.

## 2. Results

### 2.1. Effect of Temperature and Concentration Time of Progressive Freeze Concentration towards Saponin Concentration for the Initial PFC Sample

**Figure 1:**
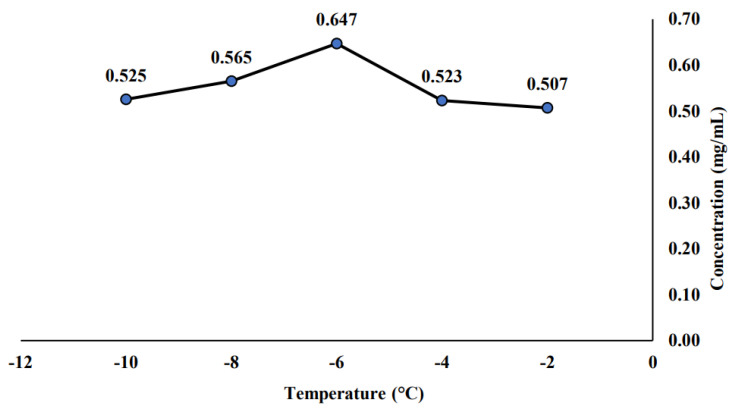
Saponin concentration of initial PFC sample at different temperatures.

**Figure 2:**
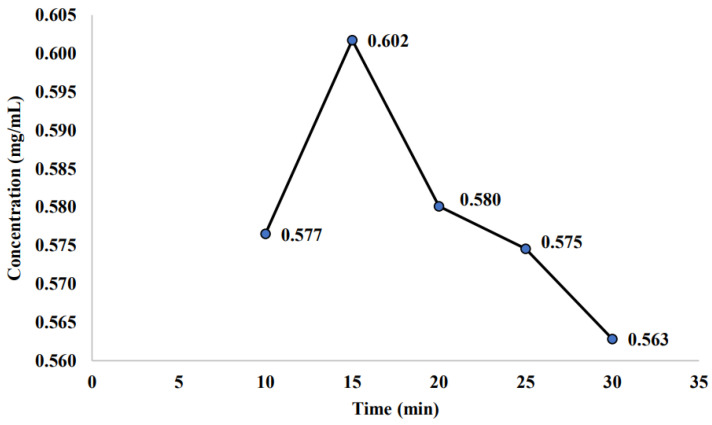
Saponin concentration of initial PFC sample at different concentration times.

In this subsection, the measured saponin concentration for the initial PFC sample was presented. The initial PFC sample is the sample obtained right after it undergoes the PFC process. These samples were not experiencing any thermal treatment or kept for 21 days in a cold room or a room temperature room. Figure 1 is constructed based on the saponin concentration observed for each sample at a different temperature of −2, −4, −6, −8, and −10 °C. The highest saponin concentration (0.647 mg/mL) is at a temperature of −6 °C, while the lowest saponin concentration is found at the highest temperature applied (−2 °C) with a value of 0.507 mg/mL. On the other hand, Figure 2 illustrated that the saponin concentration at a constant temperature (−6 °C) with the concentration time was manipulated at 10, 15, 20, 25, and 30 min for the initial PFC sample. It was observed that the highest saponin concentration has shown at a time of 15 min, while the lowest saponin concentration was measured at the highest concentration time (30 min) with a value of 0.563 mg/mL.

### 2.2. Stability Analysis

#### 2.2.1. Effect of Room Storage on the Stability of Non-Sterilized Sample

**Figure 3:**
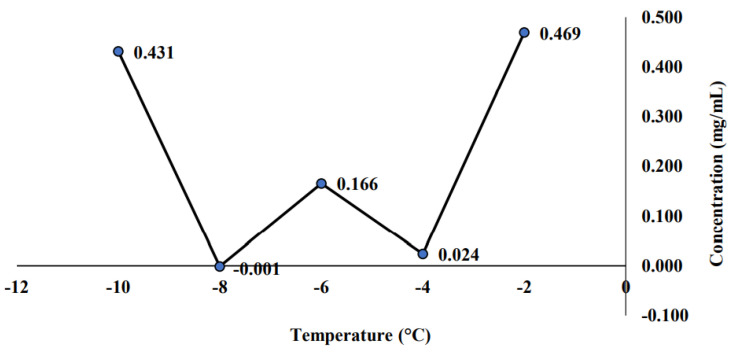
Saponin concentration of non-sterilized sample at different temperatures.

**Figure 4:**
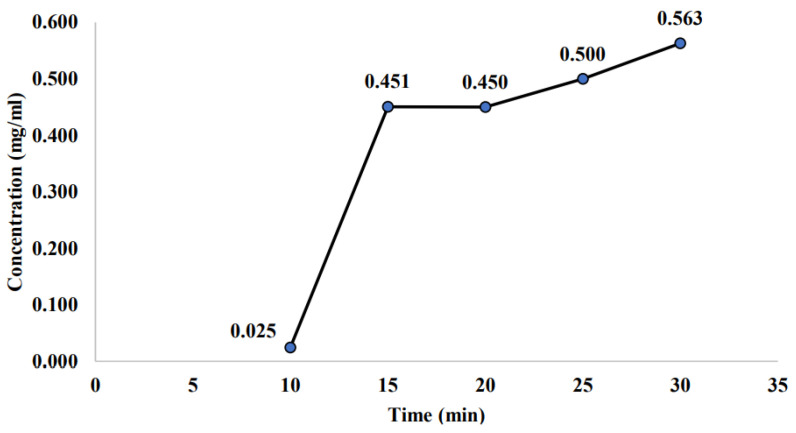
Saponin concentration of non-sterilized sample at different concentration times.

In this subsection, the measured saponin concentration for the non-sterilized sample was presented. The non-sterilized sample is the sample stored for 21 days at room temperature, and these samples were not undergoing any thermal treatment. Figure 3 displayed that the highest saponin concentration (0.469 mg/mL) was observed at the highest temperature (−2 °C). This finding is inversely related to the previous Section 2.1 that measured the initial PFC sample. Previously, the optimum temperature was shown at −6 °C with the highest saponin concentration of 0.647 mg/mL. In this case, the highest saponin concentration found for the non-sterilized samples were slightly lower than the initial PFC samples. Meanwhile, Figure 4 demonstrated the trend of saponin concentration versus concentration time. The trend showed that the saponin concentration was gradually increased as the concentration time increases. The saponin concentration rises from 0.025 to 0.563 mg/mL as the concentration time increases from 10 to 30 min. This finding again showed inconsistent results with previous findings in Section 2.1, which measured the initial PFC sample. Previously, the most suitable concentration time for saponin concentration was shown at 15 min, with the highest saponin concentration of 0.602 mg/mL. The highest saponin concentration found in this case for non-sterilized sample was slightly lower than the initial PFC sample.

#### 2.2.2. Effect of Cold Storage on the Stability of Sterilized Sample

**Figure 5:**
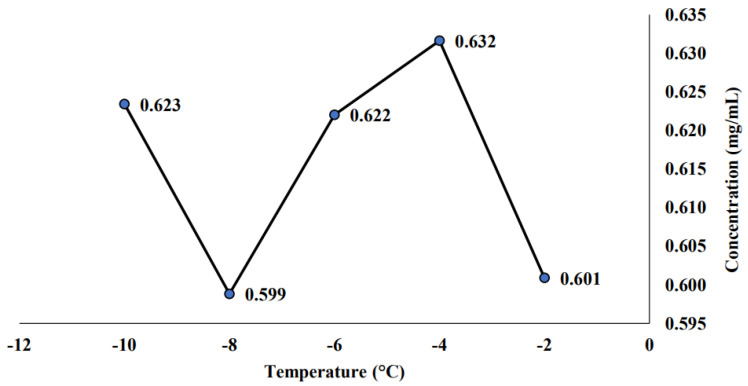
Saponin concentration of sterilized sample at different temperatures.

In this subsection, the measured saponin concentration for a sterilized sample was presented. The sterilized sample is obtained after being stored for 21 days in a cold room at 10 °C. This sample undergoes thermal treatment where the sample was poured into a 25 mL vial heated at 90 °C for 1 min in a water bath before storing them into the cold room. Figure 5 expressed that the highest saponin content (0.632 mg/mL) was found at −4 °C. The saponin concentration at different temperatures is higher than the saponin concentration of initial PFC samples (without storing and unsterilized) and final PFC samples (stored at room temperature and unsterilized). Meanwhile, the lowest saponin concentration was found at a temperature of −8 °C with 0.599 mg/mL value. Nevertheless, this value is still higher than the previous finding that measured the non-sterilized PFC sample, where the highest saponin concentration (0.469 mg/mL) was found at −2 °C.

**Figure 6:**
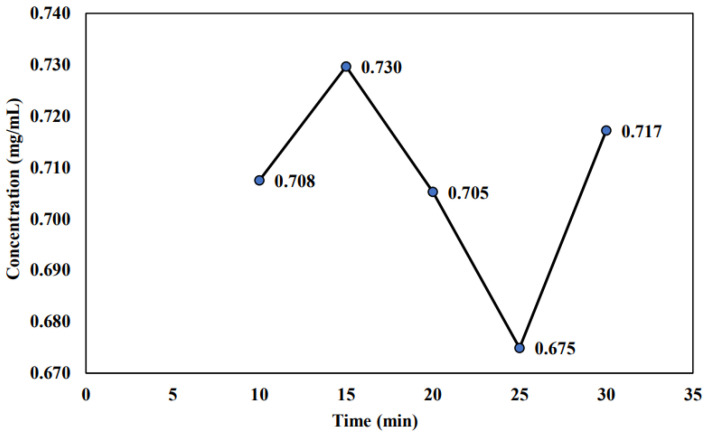
Saponin concentration of sterilized sample at different concentration times.

The highest saponin concentration was found for a 15 min concentration time with a value of 0.730 mg/mL, as shown in Figure 6. This finding is similar to the result found in the previous Section 2.1, which measured the initial PFC sample. Previously, from the finding with the initial PFC sample, the most suitable concentration time for saponin concentration was found at 15 min, with the highest saponin concentration of 0.602 mg/mL. Similarly, a similar concentration time was found but with a higher saponin concentration (0.730 mg/mL). This finding aligned with the claim that applying thermal treatment as a sterilization method could prevent the sample from degradation. Hence, this study would choose the best operating condition for the PFC sample at a temperature of −4 °C and concentration time of 15 min, which exhibits the highest saponin concentration with the value of 0.632 and 0.730 mg/mL, respectively.

## 3. Discussion

### 3.1. Effect of Temperature and Concentration Time of Progressive Freeze Concentration towards Saponin Concentration for the Initial PFC Sample

Anuar et al. [43] mentioned that the operating temperature in PFC was an important parameter as it is highly related to the freezing rate. Most of the time, the low operating temperature corresponds to a high freezing rate. As mentioned in the previous Section 2.1, the highest saponin concentration was found at a temperature of −6 °C. Hence, this finding would suggest that −6 °C is the most optimal temperature for the saponin concentration used in this study. In addition, the lowest saponin concentration was found at both the highest (−2 °C) and lowest (−10 °C) temperatures applied with a value of 0.507 and 0.525 mg/L, respectively. It was reported that the high temperature caused several saponin structures to be destroyed [44]. A similar trend was also shown in other applications where Yahya et al. [45] observed that the iodine value for olein increases as the PFC operating temperature decreased from 29 to 24 °C. They claimed that as the more soluble solids remain concentrated at a low operating temperature, the higher purity of ice solids would be achieved. 

Meanwhile, it was also claimed that at the lowest temperature, the saponin could not dissolve the saponin in the solution fully [44]. A similar finding was reported by Mazli et al. [46], where the highest oil and grease removal obtained by PFC was at a low or moderate operating temperature. The performance of PFC in terms of oil and grease removal gradually reduced as the PFC operating temperature decreased from −6 to −14 °C. Hence, in this study, the operating temperature of −6 °C had been selected as the most optimal temperature for saponin concentration of the initial PFC sample. 

In terms of concentration time, as found earlier (refer to Section 2.1), this study proposed that the most suitable concentration time for saponin was at 15 min. Prolonging the concentration time would lead to a decrement in saponin concentration. The lowest saponin concentration was measured at the highest concentration time (30 min) with a value of 0.563 mg/mL. Liu et al. [44] claimed that the higher concentration time would decrease the efficiency of the saponin extraction process and lead to an increased cost due to the fact that more energy was consumed as the concentration time increases. Hence, this study would conclude that the best operating condition for the initial PFC sample is −6 °C and the concentration time of 15 min, which exhibit the highest saponin concentration with the value of 0.647 and 0.602 mg/mL, respectively.

Nevertheless, contradicting with this finding, Anuar et al. [43] reported that for the application of oil recovery, the maximum oil recovery obtained (92.56%) was found at the longer PFC operating time (50 min). They claimed that the highest purity of solids could be achieved by prolonging the duration of the PFC process. Azman et al. [47] showed a similar finding, where the highest solute recovery (96%) was observed at the PFC operating time of 50 min. This statement was supported by Safiei et al. [48], where the higher concentration efficiency would be obtained at a longer PFC operating time. In addition, Amran et al. [49] mentioned that the higher efficiency received at the longer PFC operating time as a higher solute concentration in the concentrate would be produced. 

### 3.2. Effect of Room Storage on the Stability of Non-Sterilized Sample

As mentioned before in Section 2.2.1, the highest saponin concentration found for the non-sterilized samples was slightly lower than the initial PFC samples. This might be due to the sample degradation as they were kept at room temperature without undergoing any thermal treatment (sterilization). Hence, the sample was suggested to be sterilized to maintain the sample content and reduce sample degradation. People often mistook sterilization, cleaning, and disinfection as the same thing [50]. However, all of them are three different processes. By definition, sterilization is a process of killing or removing all organisms. Meanwhile, cleaning is a process of reducing the number of organisms present. The last one is disinfection, which removes most pathogenic organisms (organisms that could cause disease). In this study, the sterilization method used is to remove all the organisms that may cause the sample to be degraded. According to McKeen [51], under sterilization conditions or sterility, all organisms and their germinative elements (for example, eggs, spores, and endospores) are eliminated. This condition is absolute where there is no such process known as partially sterile. The sterilization process can be divided into chemical sterilization, radiation sterilization, and high temperature or pressure sterilization. This study used high temperature sterilization, known as a thermal treatment for the sterilization process.

As for the concentration time, the saponin concentration rises from 0.025 to 0.563 mg/mL as the concentration time increases from 10 to 30 min. This finding supports the claim that sample degradation happened when the samples were kept at room temperature without undergoing any thermal treatment (sterilization). Hence, the sample was suggested to be sterilized to maintain the sample content and reduce sample degradation. As mentioned previously, sterilization was able to remove all organisms, including their germinative elements, could avoid the sample from degradation, as well as maintain the sample content from any contamination that is caused by the surrounding [51]. Therefore, this study would decide that the best operating condition for the PFC sample is at a temperature of −6 °C and concentration time of 15 min, which exhibit the highest saponin concentration with the value of 0.647 and 0.602 mg/mL, respectively, without storing at room temperature to avoid any sample degradation.

### 3.3. Effect of Cold Storage on the Stability of Sterilized Sample

From Section 2.2.1, the saponin concentration at different temperatures is higher than the saponin concentration of the initial PFC samples (without storing and unsterilized) and final PFC samples (storing at room temperature and unsterilized). This finding is aligned with the previous claim made in the previous Section 3.2, where the sample needs to be sterilized to maintain the sample content and reduce the possibility of sample degradation. This is actually to ensure that all organisms, including their germinative elements, were being eliminated. Hence, no contamination would be affected by the sample [51]. 

In addition, the thermal treatment such as sterilization and keeping in the cold room to avoid any sample degradation will prevent the degradation. This condition can be denoted as double protection where the sample first needs to undergo thermal treatment for sterilization purposes (to ensure no contamination occurred towards the sample). After that, the sample was kept in the cold room for storage purposes (to ensure that the sample could maintain its content and condition throughout the storage time). Hence, it was proven that the best operating condition for the PFC sample is at a temperature of −4 °C with a concentration time of 15 min, undergoes thermal treatment, and is stored in the cold room. Both the selected temperature (−4 °C) and concentration time (15 min) were due to the highest saponin concentration obtained at the respective conditions (0.632 and 0.730 mg/mL).

## 4. Materials and Methods

### 4.1. Materials and Reagents

*Furcraea gigantea var. striata* leaves were purchased from a nursery in Perak, Malaysia. The feedstock pre-treatment was prepared by drying the feedstock at 50 °C [39] for 24 h until the moisture content is ±10% before pounding them using pestle mortal into a more refined state. Avantis Laboratory Supply supplied ethanol, phosphoric acid, acetonitrile, sulfuric acid, and p-anisaldehyde.

### 4.2. Extraction by Maceration

Dried leaves and extraction solvent (distilled water) were stirred on a hot plate with a magnetic bar for 3 h at 50 °C with leaves/solvent ratio of 3 g/400 mL (mass per volume). The magnetic stirrer revolution per minute (rpm) was set at 100 rpm to distribute the heat during the extraction. The top of the beaker was covered with aluminium foil with small holes on them. Next, the leaves residues were filtered out using a sieve. Lastly, the filter paper was used to further filter out the tiny leaves. This procedure was repeated another nine times.

### 4.3. Concentration by the Progressive Freeze Concentration

**Figure 7:**
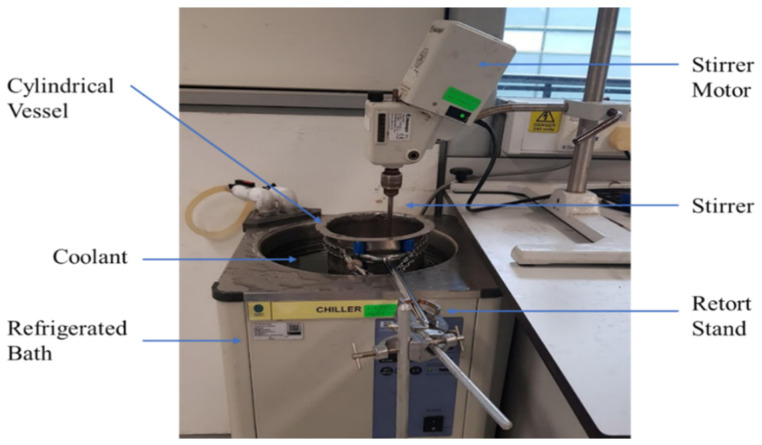
Progressive freeze concentration setup.

The experiment was setup as shown in Figure 7. Four hundred millilitres of the extract solution with distilled water as the solvent was placed in a cylindrical vessel (13.5 cm × 17 cm). The refrigerated bath was set and controlled as the temperature of the coolant (ethylene glycol + water). The extract solution was stirred by a stirrer (EURO-ST 40 D S002, IKA Works Asia Sdn Bhd, Rawang, Selangor, Malaysia) with three blades, positioned at 5 cm from the vessel’s bottom. The stirring speed was set at 75 rpm, and the concentration process was carried out for 10 min. By this configuration, the vessel allowed the formation of ice crystal only on the side of the wall.

The concentrated solution was taken and measured at the end of the desired time. The ice crystal was separated from the cooling surface of the vessel and allowed to melt entirely at room temperature. Different operating conditions such as the influence of varying coolant temperatures (−2, −4, −6, −8, and −10 °C) and concentration time (10, 15, 20, 25, and 30 min) took place on the process, the entire procedure was repeated from the beginning. To study the effect of coolant temperature, the concentration time was set to constant at 20 min, while to evaluate the impact of concentration time, the coolant temperature was fixed at −6 °C.

### 4.4. Sterilization by Thermal Treatment

The concentrated solution from the PFC process was divided into two parts: Original sample and sample of sterilization for the stability analysis. The stability analysis was conducted for two sets of experiments (non-sterilized and sterilized samples), where each set consists of five samples. Non-sterilized and sterilized samples were kept for 21 days at room temperature (around 26 °C) and in the cold room (around 10 °C), respectively. The samples kept in the cold room were the sterilized samples that undergo thermal treatment.

The original sample was poured into 25 mL vials and stored for 21 days at room temperature. Meanwhile, the sample for sterilization was poured into 25 mL vials to sterilize the sample and was kept for 21 days in a cold room (10 °C). The sample of sterilization undergoes thermal treatment before being stored in the cold room. The thermal treatment was performed using a laboratory water bath where the sample was placed on a rack to avoid direct heating from the base of the water bath. The sample was heated at 90 °C for 1 min before storing them in the cold room at 10 °C. 

## 5. Conclusions

The findings from this study showed that the highest saponin concentration for initial PFC, non-sterilized and sterilized samples was found at the temperature of −6, −2, and −4 °C, with saponin concentration of 0.647, 0.469, and 0.632 mg/mL, respectively. Meanwhile, the highest saponin concentration for the initial PFC and the sterilized sample was found at the concentration time of 15 min with the value of 0.602 and 0.730 mg/mL, respectively. Nevertheless, the highest saponin concentration found for the non-sterilized sample was at 30 min with the value of 0.563 mg/mL. In terms of stability analysis, the results showed that sterilized saponin stored in a cold room has a low degradation rate since the highest saponin concentration (0.730 mg/mL) was found compared to non-sterilized saponin stored at room temperature (26 °C). Thus, the finding concluded that saponin is sensitive to temperature. Hence, the best storage method to store saponin is after undergoing thermal treatment and being kept in the cold room at 10 °C.

## Data Availability

The data presented in this study are available within the article.
